# NagR_Bt_ Is a Pleiotropic and Dual Transcriptional Regulator in *Bacillus thuringiensis*

**DOI:** 10.3389/fmicb.2018.01899

**Published:** 2018-09-11

**Authors:** Zhang-lei Cao, Tong-tong Tan, Yan-li Zhang, Lu Han, Xiao-yue Hou, Hui-yong Ma, Jun Cai

**Affiliations:** ^1^Department of Microbiology, College of Life Sciences, Nankai University, Tianjin, China; ^2^Key Laboratory of Molecular Microbiology and Technology, Ministry of Education, Tianjin, China; ^3^Tianjin Key Laboratory of Microbial Functional Genomics, Tianjin, China

**Keywords:** N-acetylglucosamine, NagR_Bt_, pleiotropic, repressor, activator, *Bacillus thuringiensis*

## Abstract

NagR, belonging to the GntR/HutC family, is a negative regulator that directly represses the *nagP* and *nagAB* genes, which are involved in GlcNAc transport and utilization in *Bacillus subtilis*. Our previous work confirmed that the chitinase B gene (*chiB*) of *Bacillus thuringiensis* strain Bti75 is also negatively controlled by YvoA_Bt_, the ortholog of NagR from *B. subtilis*. In this work, we investigated its regulatory network in Bti75 and found that YvoA_Bt_ is an N-acetylglucosamine utilization regulator primarily involved in GlcNAc catabolism; therefore YvoA_Bt_ is renamed as NagR_Bt_. The RNA-seq data revealed that 27 genes were upregulated and 14 genes were downregulated in the Δ*nagR* mutant compared with the wild-type strain. The regulon (exponential phase) was characterized by RNA-seq, bioinformatics software, electrophoretic mobility shift assays, and quantitative real-time reverse transcription PCR. In the Bti75 genome, 19 genes that were directly regulated and 30 genes that were indirectly regulated by NagR_Bt_ were identified. We compiled *in silico, in vitro*, and *in vivo* evidence that NagR_Bt_ behaves as a repressor and activator to directly or indirectly influence major biological processes involved in amino sugar metabolism, nucleotide metabolism, fatty acid metabolism, phosphotransferase system, and the Embden–Meyerhof–Parnas pathway.

## Introduction

N-Acetylglucosamine (GlcNAc) is a nitrogen-containing monosaccharide that is a preferred nutrient source for the growth and development of many microorganisms because it is highly abundant and provides both carbon and nitrogen (Mobley et al., 1982). After being absorbed, GlcNAc is funneled into the glycolysis shunt pathway for catabolism or directed to peptidoglycan synthesis for anabolism (Bertram et al., 2011). GlcNAc is exploited for both catabolic and anabolic purposes; therefore, its correct utilization requires rigorous control.

DasR (deficient in aerial hyphae and spore formation), a member of the gluconate operon repressor (GntR) family transcriptional repressor, was initially reported to be related to the complex morphological differentiation in *Streptomyces griseus* (Seo et al., 2002). Subsequently, Rigali et al. found the role of DasR in GlcNAc metabolism using *Streptomyces coelicolor* as a model organism (Rigali et al., 2004). Later studies revealed that DasR is a global regulator that plays a pivotal role in the regulation of antibiotic synthesis, morphological differentiation, and GlcNAc transport and metabolism (Rigali et al., 2006, 2008; Swiatek et al., 2012; Tenconi et al., 2015). In addition, it had also been shown to act as a repressor and an activator in *S. coelicolor* (Nazari et al., 2013) (Swiatek-Polatynska et al., 2015). The DNA-binding site of DasR has been identified as a conserved 16-bp consensus sequence in *S. coelicolor*, and was named the DasR-responsive element (*dre*) (Colson et al., 2007). DasR can specifically bind the *dre* sites in the upstream regions of some genes to control their expressions.

Bertram et al. found that YvoA, an ortholog of DasR, is a less prominent regulator than DasR in *Bacillus subtilis* and only directly represses the *nagP* and *nagAB* genes, which are involved in GlcNAc transport and utilization (Bertram et al., 2011). Moreover, as the distribution of predicted YvoA-binding sites (*dre*_*Bacillus*_) was limited to *nagP* and the *nagAB-yvoA* locus within the chromosome of *B. subtilis*, they suggested renaming YvoA as NagR, for the GlcNAc utilization regulator.

In our previous work, we found that, in addition to *nagP* and *nagAB*, chitinase A and chitinase B genes are also negatively controlled by YvoA_Bt_ (Jiang et al., 2015). Since the main genes controlled by YvoA_Bt_ are involved in GlcNAc catabolism, we renamed YvoA_Bt_ as NagR_Bt_. Moreover, we predicted some other genes that might have *dre*-like sites on their promoter region or in nearby areas using the PREDetector software program. Consequently, we speculated that NagR_Bt_ might act as a pleiotropic transcriptional regulator to modulate more genes in various metabolic pathways.

In the present study, we demonstrate that NagR_Bt_ acts as a pleiotropic transcriptional regulator that controls at least 19 genes directly and 30 genes indirectly, and these genes are involved in amino sugar metabolism, nucleotide metabolism, fatty acid metabolism, phosphotransferase system, and the Embden–Meyerhof–Parnas (EMP) pathway. Moreover, our study indicates that NagR_Bt_ is also a dual transcription regulator that acts as both a repressor and an activator in Bti75. Finally, a regulatory model network of NagR_Bt_ is presented and discussed.

## Materials and methods

### Bacterial strains and culture conditions

The wild-type *Bacillus thuringiensis* strain Bti75 and its mutants were used in this study. These strains were cultivated at 30°C in an orbital shaker (200 rpm). *Escherichia coli* DH5α, and *E. coli* BL21 (DE3) were generally grown at 37°C with shaking at 200 rpm. All the strains were cultured in lysogeny broth (LB) medium. The culture medium contained the following appropriate concentrations of antibiotics: 50 μg mL^−1^ of erythromycin (Erm), 100 μg mL^−1^ of ampicillin (Amp), 10 μg mL^−1^ of chloramphenicol (Cm), and 100 μg mL^−1^ of kanamycin (Kan). The bacterial strains and plasmids used in this study are listed in Table 1.

**Table 1:** Bacterial strains and plasmids used in this study.

**Plasmid or strain**	**Relevant characteristic(s)^a^**	**Source or reference**
**STRAINS**		
Bti75	Wide-type strain	Laboratory collection
Bti75Δ*nagR*	Bti75*ΔnagR*	Laboratory collection
Bti75Δ*nagR*-pP*erm*-*nagR*	Bti75*ΔnagR*-pP*erm*-*nagR*	This study
*E. coli* DH5α		Laboratory collection
*E. coli* BL21-pET- *nagR*	Kan^r^; *nagR* gene cloned into pET-28a(+), His tag binding C terminus	Laboratory collection
**PLASMIDS**		
pKSV7	Amp^r^ Em^r^ Cm^r^; *Bacillus-E. coli* shuttle vector, temp sensitive	Laboratory collection
pKSV7-P*erm*-*nagR* (pP*erm*-*nagR*)	Amp^r^ Em^r^ Cm^r^; 732bp fragment of *nagR* overlapped with the promoter of *erm* cloned into the pKSV7 *Eco*RI/*Xba*I site	This study
pCB	Amp^r^ Em^r^; the purpose promoter-probe vector containing the β-galactosidase gene *bgaB*; *Bacillus-E. coli* shuttle vector, temp sensitive	Laboratory collection
pCB-*Ppgi*	Amp^r^ Em^r^; the promoter of *pgi* without its *dre* site cloned into the pCB *Bam*HI/*Sa*lI site	This study
pCB-*dre*+P*pgi*	Amp^r^ Em^r^; the promoter of *pgi* with its *dre* site cloned into the pCB *Bam*HI/*Sa*lI site	This study
pCB-P*pgi*+*dre*	Amp^r^ Em^r^; the position interchange between the promoter of *pgi* and its *dre* site cloned into the pCB *Bam*HI/*Sal*I site	This study

a *Amp^r^, ampicillin resistance; Em^r^, erythromycin resistance; Cm^r^, chloramphenicol resistance; Kan^r^, kanamycin resistance*.

### Construction of *nagR* deletion mutants and complemented strains

The *nagR* (formerly termed *yvoA*) deletion mutant, Δ*nagR*, was constructed in our laboratory and reported previously (Jiang et al., 2015). To construct the complemented mutant strain of *nagR*, the fragment of *nagR* was amplified from Bti75. The fragment was overlapped with the *erm* promoter, digested with *Eco*RI and *Xba*I, and inserted into the corresponding sites of pKSV7, to create the pKSV7-P*erm*-*nagR* (pP*erm*-*nagR*) complementing plasmid. The resulting plasmid was transferred into Bti75 by electroporation (Lecadet et al., 1992). The desired complementary strains were screened by Cm-resistance screening, PCR detection, and sequencing verification. All primers used in this study are shown in Table S1.

### Electrophoretic mobility shift assay

The expression and purification of NagR_Bt_ (formerly termed YvoA_Bt_) were performed according to previously described methods (Jiang et al., 2015). DNA probes (16 bp) obtained using software prediction were extended by 12 bp on both sides. The ~40 bp DNA probes were generated by annealing primers. The DNA probes were heated at 98°C for 5 min and then incubated at room temperature for 20 min. The upstream regions of each differentially expressed gene (~300 bp upstream of the ATG start site) from RNA-seq were generated using PCR reactions. The 5′ ends of some primers were labeled with biotin for non-specific and specific competition assays. The concentrations of proteins and DNA probes were determined using a NanoDrop 2000 spectrophotometer (NanoDrop Technologies, Wilmington, DE, USA) and are indicated in the corresponding figures.

Electrophoretic mobility shift assays (EMSAs) were carried out at 30°C for 30 min in a binding reaction buffer containing 10 mM Tris HCl (pH 8.0), 1 mM MgCl_2_, 50 mM NaCl, 0.5 mM DTT, 0.5 mM EDTA, and 5% glycerol. After binding, the samples were separated using a non-denaturing polyacrylamide gel in an ice bath of 1 × Tris-borate-EDTA (TBE) buffer at 50 V for 4 h. Then the gel was exposed to ultraviolet light after EB staining for preliminarily screening. For DNA probes, which gave an obviously shifted band in the direct assay, unlabeled specific fragments (400-fold) and non-specific competitor DNA (0.5 mg mL^−1^ sheared salmon sperm DNA) were used. In this case, biotin-labeled probes were incubated with NagR_Bt_. After binding, the samples were loaded and separated on non-denaturating PAGE gels, and then transferred onto a nylon membrane. The biotin-labeled probes were detected using a biotin chromogenic detection kit (Thermo Fisher Scientific Inc.).

### RNA preparation and transcriptome assay

Starting with an independent colony from the LB agar plate, Bti75 and Bti75 Δ*nagR* were grown overnight in LB medium at 30°C. They were diluted 1:100 (vol/vol) into LB and shaken at 200 rpm at 30°C for ~9 h, at which point they reached the exponential phase. The bacteria were harvested, immediately frozen in liquid nitrogen, and stored at −80°C until use. The two samples (Bti75 and Bti75 Δ*nagR*) were shipped to GENEWIZ (www.genewiz.com) for mRNA-seq library construction and transcriptome sequencing. The mRNA-seq library construction comprised the following steps: RNA extraction and quality control (Agilent Eukaryote Total RNA Nano Kit, Agilent 2100), library construction (NEBNext Ultra RNA Library Prep Kit), library purification (Beckman AMPure XP beads), library detection (Agilent High Sensitivity DNA Kit, Agilent 2100), quantitative analysis of the library (ABI 7500 RealTime PCR System, KAPA SYBR Green FAST Universal 2 × qPCR Master Mix), and cBOT automatic clusters (TruSeq PE Cluster Kit v3). The process of each step was strictly controlled and then Solexa sequencing was performed using an Illumina HiSeq™ 2500 instrument for each sample that passed the quality test. The process of RNA-Seq data analysis was as follows. The original data were identified using CASAVA (v1.8.2) (Hosseini et al., 2010) and the excess adaptors and low-quality sequences were removed to obtain clean reads using Trimmomatic (v0.30) (Bolger et al., 2014). High-quality sequences were further analyzed by downstream processing and mapped according to the reference sequence (ftp://ftp.ncbi.nih.gov/genomes/Bacteria/Bacillus_thuringiensis_HD_789_uid173860/) using bowtie2 (2.1.0) (Langdon, 2015). The assembled sequences were analyzed based on a series of databases to classify and annotate the obtained reads. The normalization method of fragment per kilobase per million mapped reads (FPKM) (Mortazavi et al., 2008) was used to calculate gene expression.

### cDNA synthesis and quantitative real-time reverse transcription PCR analysis

cDNA was synthesized from the RNA of each sample using a PrimeScript™ RT Reagent Kit with gDNA Eraser (Takara, Dalian, China), according to the manufacturer's instructions. Quantitative real-time reverse transcription PCR (qRT-PCR) was performed using TB Green *Premix Ex Taq* II (Takara) and the PCR products were detected using a StepOnePlus Real-Time PCR System (Applied Biosystems, Foster City, CA, USA) based on the manufacturer's instructions. The relative expression level of each gene was normalized by that of 16S rRNA, as an endogenous control, and calculated according to the 2^−ΔΔ*CT*^ method (Livak and Schmittgen, 2001). Experiments using independent biological and technical replicates of each biological sample for each gene were repeated three times to ensure reliability and reproducibility. The primers for qRT-PCR are presented in Table S1.

### Promoter/*dre* position swapping experiments

Promoter/*dre* position swapping experiments were carried out using a promoter-probe vector pCB containing the reporter gene β-galactosidase (*bgaB*) (Xie et al., 2012). This vector was used to determine the effect of the changes in the relative position between the promoter and the *dre* site on the expression of the reporter gene. The *pgi* gene was activated by NagR_Bt_ and we chose its upstream region to construct the corresponding plasmids. Different fragments carrying the *pgi* promoter with and without the *dre* operator were amplified: 1, promoter without a *dre* “P*pgi*” (oligos P*pgi*-F and P*pgi*-R); 2, promoter with its own *dre* “*dre*+P*pgi*” (oligos P*dre*+P*pgi*-F and P*pgi*-R); 3, promoter with *dre* downstream “P*pgi*+*dre*” (oligos P*pgi*-F and P*pgi*+*dre*-R) (Figure 4A). F oligos carry *Bam*HI sites and R oligos carry *Sal*I sites. P*pgi*+*dre*-R also has an extension with a copy of the *dre* site. Fragments were digested with *Sal*I and *Bam*HI enzymes and inserted into pCB digested with the same enzymes creating pCB-P*pgi*, pCB-*dre*^*pgi*^+P*pgi*, and pCB-P*pgi*+*dre*^*pgi*^, respectively. The resulting plasmids were transferred into Bti75 using electroporation, and then the expression of *bgaB* was determined using qRT-PCR.

### β-galactosidase activity assay

β-galactosidase activity of Bt strains were performed according to the protocols as previously described (Hirata et al., 1984) and expressed in Miller units. All the reactions were run at least in three independent assays.

### Computational prediction of *dre* sequences in Bti75

NagR_Bt_-binding upstream sequences of five *dre* targets (*dre*^*nagA*^: GCACGAGTAGTTGTCT; *dre*^*nagP*^: ACACATCTATACAACT; *dre*^*chiB*^: AGACTTCGTGATGTCT; *dre*^*chiA*^: ATACATCTAGACAACT; *dre*^*chitin*^: AGTTGGCTAGTCATCT) in Bti75 were used to predict the NagR_Bt_-binding sites in *B. thuringiensis* subsp. *israelensis* strain HD-789 using PREDetector (Hiard et al., 2007). The position weight matrix fitted out by these five binding sequences was used to predict the potential *dre* sites, which are listed in Table S2; the cutoff score was greater than 7.0.

## Results

### Transcriptome sequencing

To study the regulatory range of NagR_Bt_, we used Solexa/Illumina sequencing to perform a transcriptome sequencing analysis for two samples (Bti75 and Bti75-Δ*nagR*) in the exponential phase (~9 h). In the gene expression analysis, a mathematic algorithm was used to identify differentially expressed genes [false discovery rate (FDR) < 0.01 and absolute log_2_ values ratio ≥ 2]. This analysis identified 27 upregulated genes and 15 downregulated genes in Bti75-Δ*nagR*. The RNA-seq data were deposited at the NCBI Sequence Read Archive (submission no. SUB2391258).

As shown in Table 2, data from the RNA-seq analysis revealed that the expression of genes *nagAB, nagP, chiA, chiB*, and chitin-binding protein were all increased in the Δ*nagR* mutant relative to the wild-type strain. These genes, which are involved in amino sugar metabolism, were previously shown to have a *dre* site. In addition, we found other differentially expressed genes (DEGs) in Bti75-Δ*nagR*. Cytidine deaminase (BTF1_06850), pyrimidine-nucleoside phosphorylase (BTF1_06845), and pyrimidine nucleoside transporter (BTF1_06840) involved in nucleotide metabolism might be on the same operon. The expression of genes, lipoprotein (BTF1_26870), acyl-dehydrogenase (BTF1_25115 and BTF1_08960), and acyl carrier protein (BTF1_17540) involved in fatty acid metabolism were decreased in the Bti75-Δ*nagR* mutant, suggesting that NagR_Bt_ could activate these genes, rather than repress them. Glucose-specific phosphotransferase enzyme IIA (BTF1_24980), phosphoenolpyruvate-protein phosphotransferase (BTF1_18555), phosphocarrier protein HPr (BTF1_18560), and the PTS lactose cellobiose family IIC subunit (BTF1_24425) involved in the phosphotransferase system showed enhanced expression as previously reported in *S. coelicolor* (Rigali et al., 2004, 2006).

**Table 2:** Genes controlled by NagR_Bt_ in Bti75.

**Locus_tag^a^**	**Gene**	**Description**	**Positions^b^**	***dre* motif^c^**	**Score^d^**	**Fold change^e^**	**qRT-PCR^f^**
**GENES UPREGULATED IN THE** *Δ**nagR*** **MUTANT COMPARED TO WILD-TYPE STRAIN Bti75**							
BTF1_00220	*nagP*	PTS system subunit IIBC	−107	acacatctatacaact	12.12	17.301	21.967 ± 0.785
BTF1_01610		Outer surface protein	−156	agatgtatagacttgt	9.8	20.046	6.046 ± 0.280
BTF1_01615	*licR*	BigG family transcription antiterminator	/	/	/	7.751	NT
BTF1_06840	*nupC*	nucleoside transporter NupC	/	/	/	4.738	NT
BTF1_06845	*pdp*	Pyrimidine-nucleoside phosphorylase	/	/	/	14.614	NT
BTF1_06850	*cdd*	Cytidine deaminase	/	/	/	19.753	45.010 ± 2.977
BTF1_07625		Hypothetical protein	/	/	/	12.407	NT
BTF1_09020	*oxlT*	Oxalate formate antiporter	/	/	/	10.024	NT
BTF1_11065		Hypothetical protein	/	/	/	12.690	NT
BTF1_11665	*lpmo*	Chitin-binding protein	−56	agttggctagtcatct	9.56	25.204	NT
BTF1_16665	*chiA*	Extracellular exochitinase Chi36	−36	atacatctagacaact	12.76	6.679	3.551 ± 0.270
BTF1_18555	*ptsI*	Phosphoenolpyruvate-protein phosphotransferase	/	/	/	6.284	NT
BTF1_18560	*ptsH*	Phosphocarrier protein HPr	−52	agttgtatagacgtga	9.82	6.395	4.716 ± 0.299
BTF1_18585	*nagB*	Glucosamine-6-phosphate deaminase	/	/	/	11.365	NT
BTF1_18590	*nagA*	N-acetylglucosamine-6-phosphate deacetylase	−90	gcacgagtagttgtct	8.05	21.012	36.413 ± 1.955
BTF1_19070		Hypothetical protein	−78	acatgtcaagacaact	9.82	ND	NT
BTF1_19250		Hypothetical protein	/	/	/	7.169	NT
BTF1_20050	*ispH*	4-hydroxy-3-methylbut-2-enyl diphosphate reductase	/	/	/	7.307	NT
BTF1_23785	*dps*	DNA protection during starvation protein 2	/	/	/	7.428	NT
BTF1_24425	*chbC*	PTS system cellobiose-specific transporter subunit IIC	/	/	/	4.807	NT
BTF1_24980	*crr*	PTS system glucose-specific transporter subunit IIA	−251	acttgtctagaggtct	8.87	75.026	23.846 ± 1.973
BTF1_25170		XRE family transcriptional regulator	/	/	/	33.600	NT
BTF1_25685	*lrgB*	Antiholin-like protein LrgB	/	/	/	19.299	NT
BTF1_25690	*lrgA*	Murein hydrolase regulator LrgA	/	/	/	10.165	NT
BTF1_26820		Group-specific protein	/	/	/	13.357	NT
BTF1_26825	*glmS*	Glucosamine-fructose-6-phosphate aminotransferase	/	/	/	5.506	NT
BTF1_28050	*chiB*	Chitinase B	−113	agacatcacgaagtct	9.57	39.320	NT
**GENES DOWNREGULATED IN THE** *Δ**nagR*** **MUTANT COMPARED TO WILD-TYPE STRAIN Bti75**							
BTF1_00565	*pphA*	Serine/threonine protein phosphatase	/	/	/	0.0943	NT
BTF1_00720	*nprE*	Bacillolysin, neutral protein Npr599	/	/	/	0.153	0.649 ± 0.058
BTF1_04880	*liaI*	Hypothetical protein	/	/	/	0.0542	NT
BTF1_04885		PspA/IM30 family protein	/	/	/	0.0584	NT
BTF1_06780	*nheA*	Non-hemolytic enterotoxin lytic component L2	/	/	/	0.207	0.391 ± 0.056
BTF1_08960		Acyl-dehydrogenase	/	/	/	0.214	0.4480 ± 0.046
BTF1_12180		Metallo-beta-lactamase, hydrolase	/	/	/	0.133	0.335 ± 0.027
BTF1_16395		Mutt/nudix family protein	/	/	/	0.116	NT
BTF1_17540	*acpP*	Acyl carrier protein	/	/	/	0.206	NT
BTF1_18580	*nagR*	GntR family transcriptional regulator NagR	/	/	/	0.0312	NT
BTF1_19025		Hypothetical protein	−107	aatcatctagacaact	10.1	0.0187	0.570 ± 0.030
BTF1_23020	*pgi*	Glucose-6-phosphate isomerase	−168	agatgtatatacatca	7.08	0.188	0.6781 ± 0.038
BTF1_24870	*nuoC*	NADH dehydrogenase subunit C	/	/	/	0.180	0.199 ± 0.020
BTF1_25115	*mmgC*	Acyl-CoA dehydrogenase	/	/	/	0.172	NT
BTF1_26870		Lipoprotein	/	/	/	0.133	0.817 ± 0.038
**GENES WITH RECOGNIZED** ***dre*** **SITES BUT NOT DIFFERENTIALLY EXPRESSED IN** *Δ**nagR*** **MUTANT COMPARED TO WILD-TYPE STRAIN Bti75**							
BTF1_00745		Transcription antiterminator, LytR family protein	−45	atacatttagaagtgt	7.03	NS	NT
BTF1_01335		Peptidase, M23/M37 family protein	−35	agttgtatagttgtaa	7.58	NS	NT
BTF1_02575		Major facilitator superfamily protein	−133	agatatctaaataact	8.35	NS	NT
BTF1_03055		TetR family transcriptional regulator	−194	agatgtatagtcgact	10.7	NS	NT
BTF1_19055	*pepT*	Peptidase T	−116	acatgtctagataacc	8.88	NS	NT
BTF1_23845		Wall-associated protein	−276	cgtcatctatacatct	8.62	NS	NT
BTF1_24430	*chbB*	PTS system, lichenan-specific IIB component	−249	agttgtctagttatgc	9.2	NS	NT
BTF1_26160	*bofA*	SigmaK-factor processing regulatory protein BofA	−39	atacaaatagatgtat	7.19	NS	NT

a *The BTF1 number of genes are the open reading frames based on the genome annotation of B. thuringiensis HD-789 (Doggett et al., 2013)*.

b *The position of the outermost end of a dre site relative to the respective translation start point of the gene*.

c *The dre-motifs were detected after using the PREDetector software program*.

d *The scores were determined after using the PREDetector software program*.

e *Fold change gene expression is given for a gene in the ΔnagR mutant strain compared to the wild-type in RNA-seq*.

f *Fold change gene expressions are shown as the mean ± SD from three independent biological and technical replicates of each biological sample for each gene in the ΔnagR mutant strain compared to the wild-type by qRT-PCR*.

*ND, no expression was detected in wild-type strain. NS, No significant difference between wild-type and ΔnagR mutant. NT, No test. /, No result was offered by the PREDetector software program*.

**Figure 1:**
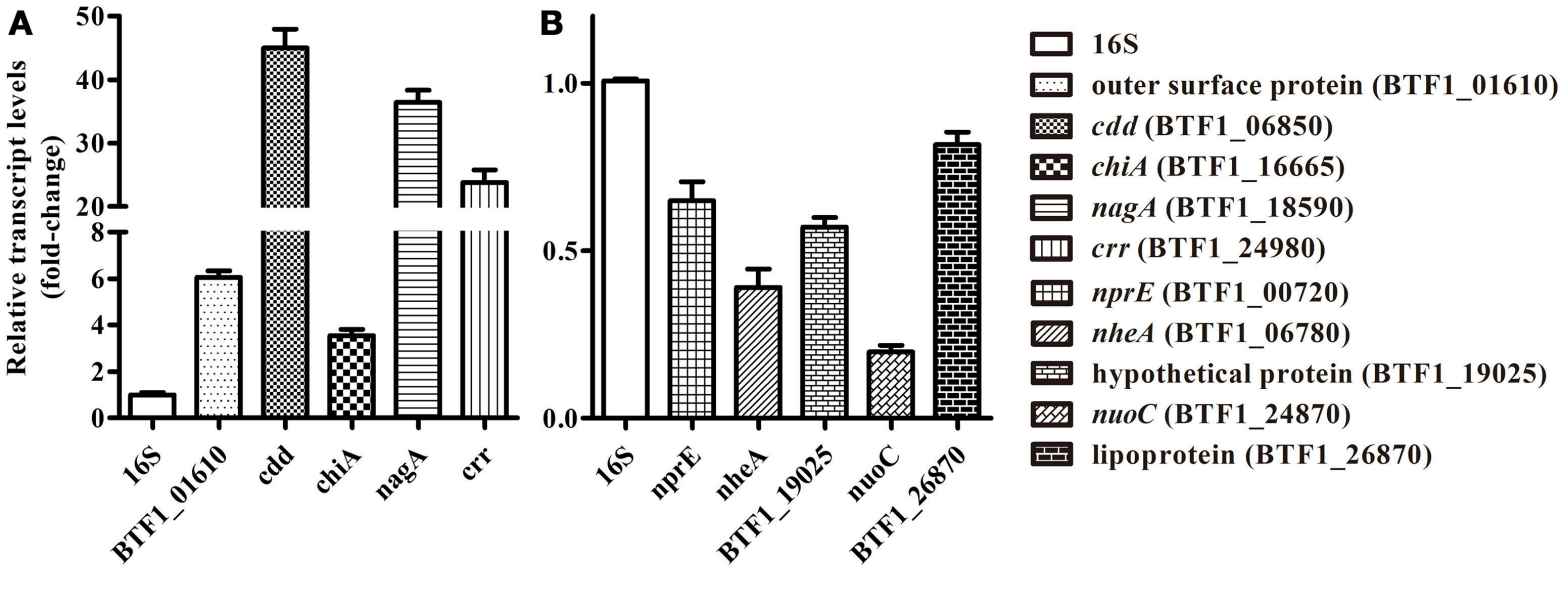
qRT-PCR validation of RNA-seq data. **(A)** Five DEGs that showed increased expression were selected randomly, namely BTF1_01610 (outer surface protein), BTF1_06850 (*cdd*), BTF1_16665 (*chiA*), BTF1_18590 (*nagA*), and BTF1_24980 (*crr*). **(B)** Five DEGs that showed decreased expression were selected randomly, namely BTF1_00720 (*nprE*), BTF1_06780 (*nheA*), BTF1_19025 (hypothetical protein), BTF1_24870 (*nuoC*), and BTF1_26870 (Lipoprotein).

To validate the reliability of the Solexa/Illumina sequencing technology, 10 DEGs were selected randomly from different metabolic pathways for qRT-PCR analysis. As shown in Figure 1, the results of the qRT-PCR analysis were consistent with those of the Solexa/Illumina sequencing analysis, although there were quantitative differences.

### Complemented mutant strain

**Figure 2:**
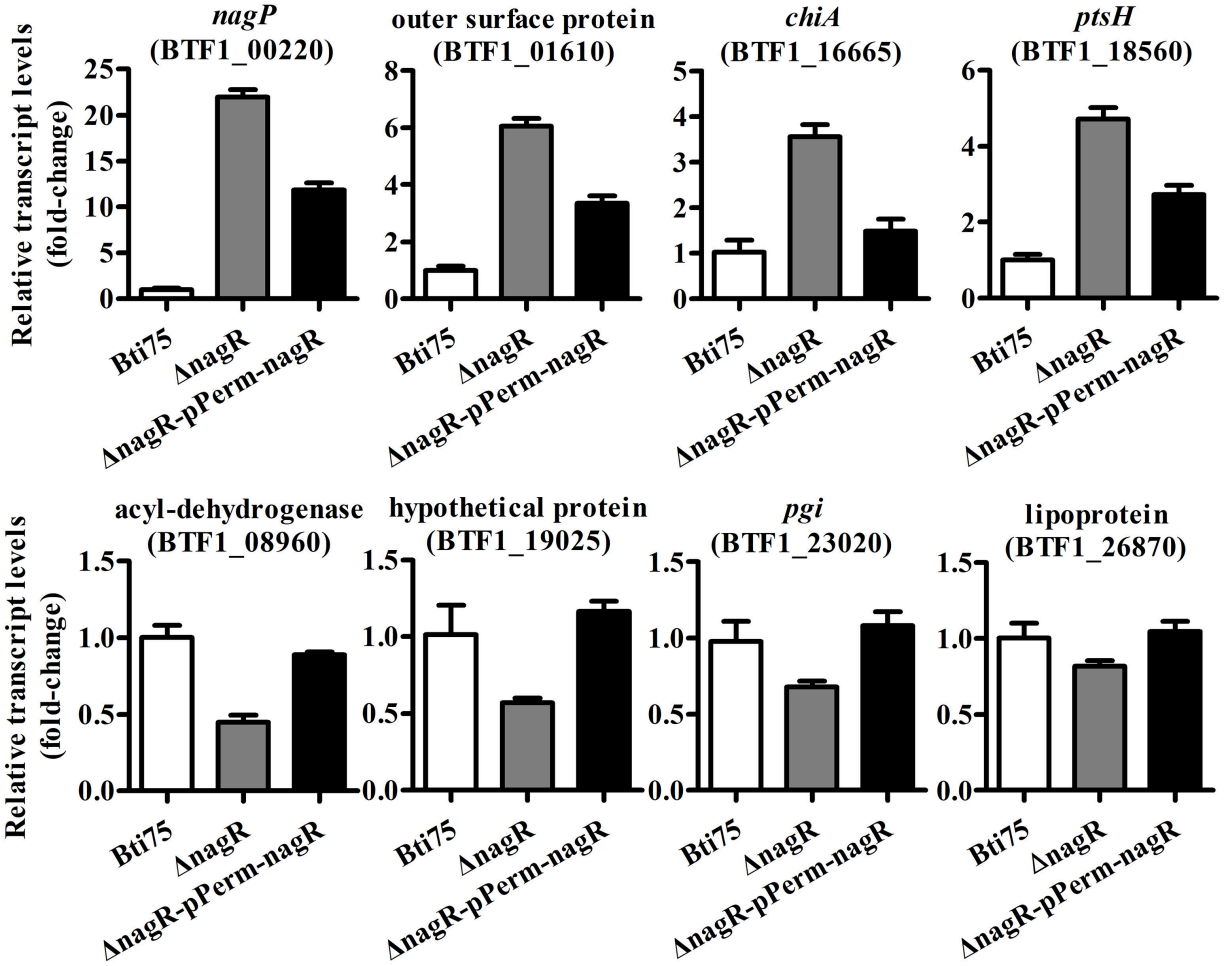
qRT-PCR validation of Bti75Δ*NagR*, Bti75Δ*nagR*-pP*erm*-*nagR*, and the wild-type (WT). Expression profiles of selected random DEGs (BTF1_00220, BTF1_01610, BTF1_16665, BTF1_18560, BTF1_08960, BTF1_19025, BTF1_23020, BTF1_26870) as determined by qRT-PCR.

The complementing vector pKSV7-P*erm*-*nagR* was constructed and its structure confirmed by PCR and restriction enzyme digestion analysis. Subsequently, the recombinant plasmid was transferred into Bti75Δ*nagR* by electroporation. The complemented clones with resistance to chloramphenicol were confirmed by PCR and DNA sequencing. The expression of gene *nagR* was detected by using qRT-PCR in Bti75Δ*nagR*, Bti75Δ*nagR*-pP*erm*-*nagR*, and the wild-type strain. The result showed that no expression of *nagR* was detected in Bti75Δ*nagR* and the fold-change of gene *nagR* was similar in the wild-type strain and Bti75Δ*nagR*-pP*erm*-*nagR* (Figure S1). Moreover, randomly selected genes were assessed using qRT-PCR to determine the influence of *nagR*. The results revealed that the fold-change of each gene in Bti75Δ*nagR*-pP*erm*-*nagR* was between the value for the wild-type and Bti75Δ*nagR*. These results suggested that changes in genes expression were indeed caused by the deletion of *nagR* (Figure 2).

### Distribution of NagR_Bt_-binding sites in the genome

To identify the regulons controlled by NagR_Bt_ in Bti75, we predicted its binding sites in the Bt HD-789 genome using PREDetector (Hiard et al., 2007). The genome of HD-789 served as the reference genome because of its high sequence similarity with Bti75. Five known *dre*-binding sites from Bti75 were used as the input to the program to construct the binding site recognition profile. Table S2 shows the output for the predicted NagR_Bt_-binding sites (67 candidates) with scores >7.0. Furthermore, compared with transcriptome data, 11 DEGs were found to harbor the predicted binding sites, implying that these genes might be regulated by NagR_Bt_ directly (Table 2).

### Identification of genes directly controlled by NagR_Bt_

All the DEGs were tested using EMSAs. If the DEG possessed a predicted *dre* site, the predicted 16 bp sequence was extended by 12 bp on both sides and the 5′ end labeled with biotin (for competition experiments) to use for EMSAs with NagR_Bt_. If not, ~300 bp of the upstream sequence from the DEG was amplified by PCR with unlabeled or labeled biotin primers.

**Figure 3:**
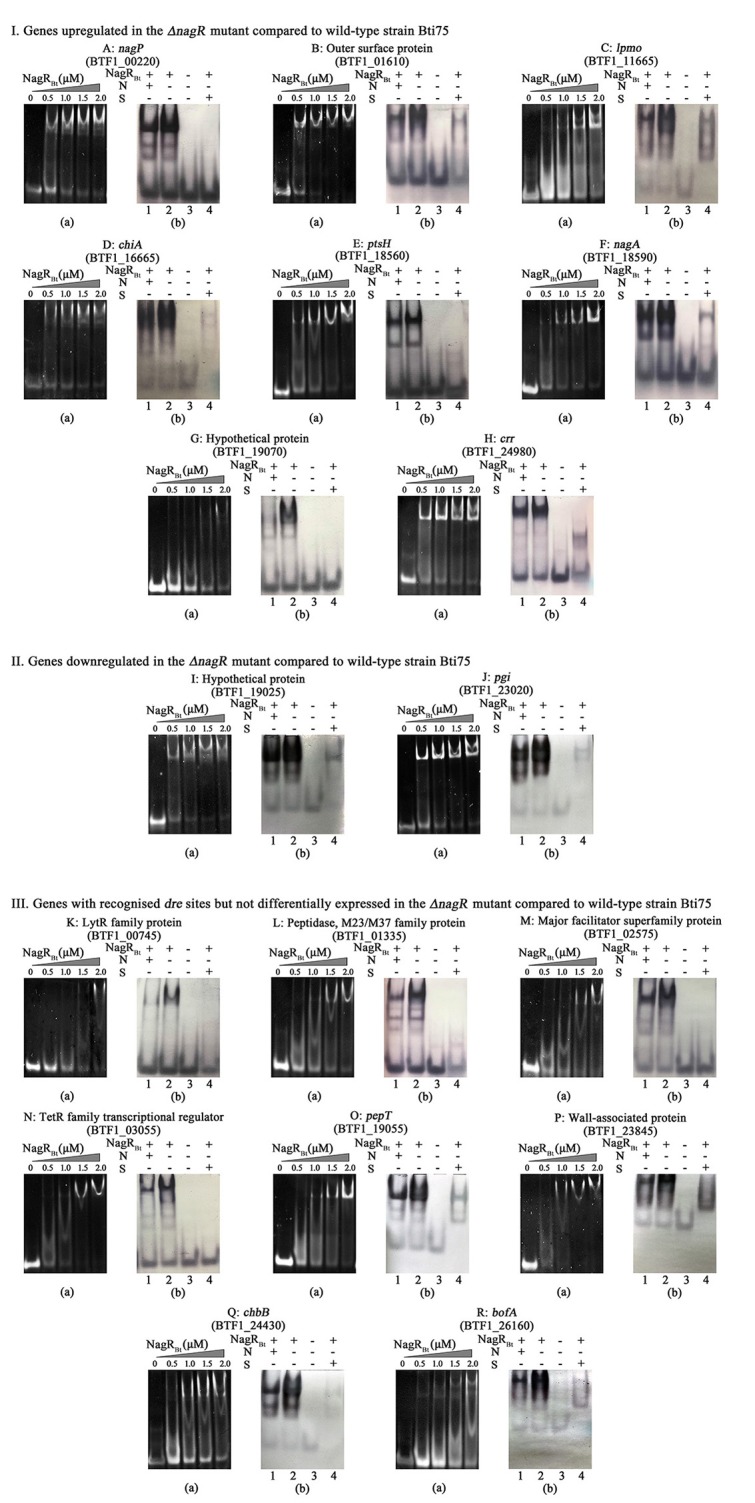
NagR_Bt_-binding sites are detected by EMSAs. **(I)** Genes upregulated in the Δ*nagR* mutant compared to wild-type strain Bti75; **(II)** Genes downregulated in the Δ*nagR* mutant compared to wild-type strain Bti75; **(III)** Genes with recognized *dre* sites but not differentially expressed in the Δ*nagR* mutant compared to wild-type strain Bti75. (a): Each 0.2 μM 40 bp-Pdre was electrophoresed after incubation with various concentrations of NagR_Bt_. (b): EMSAs to determine the specific binding of NagR_Bt_. Lane 1, Pdre (Bio), NagR_Bt_, and 0.5 μg/μL salmon sperm DNA for non-specific competitor (N); Lane 2, Pdre (Bio), NagR_Bt_; Lane 3, Pdre (Bio); Lane 4, Pdre (Bio), NagR_Bt_, and a 400-fold excess of unlabeled Pdre for specific competitor (S). All the non-specific and specific competition assays used 3.0 μM NagR_Bt_ and 0.2 μM Pdre (Bio). Different letters represent different genes. A: *nagP*, BTF1_00220; B: outer surface protein, BTF1_01610; C: *lpmo*, BTF1_11665; D: *chiA*, BTF1_16665; E: *ptsH*, BTF1_18560; F: *nagA*, BTF1_18590; G: hypothetical protein, BTF1_19070; H: *crr*, BTF1_24980; I: hypothetical protein, BTF1_19025; J: *pgi*, BTF1_23020; K: lytR family protein, BTF1_00745; L: peptidase, BTF1_01335; M: major facilitator superfamily protein, BTF1_02575; N: TetR family transcriptional regulator, BTF1_03055; O: *pepT*, BTF1_19055; P: wall-associated protein, BTF1_23845; Q: *chbB*, BTF1_24430; R: *bofA*, BTF1_26160.

In Figure 3A (DNA was detected by ethidium bromide staining), we show all the 18 positive targets whose migration was retarded by NagR_Bt_ respectively. Since the *dre* site of *chiB* has been reported by Jiang et al. (2015), it is not shown in Figure 3. To further validate the specific binding between NagR_Bt_ and *dre*, 0.5 mg mL^−1^ of salmon sperm DNA (non-specific competition) and a 400-fold excess of unlabeled *dre* sequence (specific competition) were added to the reaction mixture of NagR_Bt_ with biotin-labeled *dre* (Figure 3B, Biotin-labeled probes were detected after transfer to nylon membranes). The positive results indicated that the salmon sperm DNA failed to block the binding of NagR_Bt_ to biotin labelled *dre*, while the 400-fold excess of unlabelled *dre* sequence could eliminate or reduce the specific shift band. The results from these experiments revealed that 11 site sequences with significantly different expression could bind specifically with NagR_Bt_ and these *dre* sites were also found by computational prediction. However, the addition of NagR_Bt_ to the DNA fragments covering the 300 bp upstream of the DEG without recognizable *dre* sequences did not cause any band shift (data not shown). Furthermore, other NagR_Bt_-binding sites (from genes that did not show differential expression) from the computational prediction were detected by EMSAs and we found that seven sites could be bound by NagR_Bt_ specifically. All the different positive *dre* sequences are listed in Table 2.

### NagR_Bt_ activates the expression of some genes directly

The gene encoding a hypothetical protein (BTF1_19025) and the *pgi* (glucose-6-phosphate isomerase, BTF1_23020) gene both possess a predicted *dre* site, and NagR_Bt_ can specifically bind to them (Figures 3Ia,b,Ja,b). Most members of the GntR/HutC family are repressors, including NagR_Bt_. However, comparative transcriptome analysis showed that the expression of these two genes are downregulated rather than upregulated in the NagR_Bt_ deletion mutant (Table 2). qRT-PCR data were qualitatively consistent with NagR_Bt_ acting as a positive regulator at these two genes. BTF1_19025 and BTF1_23020 expression in Bti75Δ*nagR* decreased to ~0.57 and 0.68 of the transcription observed in the wild-type respectively (Figure 2). These results suggested that NagR_Bt_ could activate the expression of these two genes.

**Figure 4:**
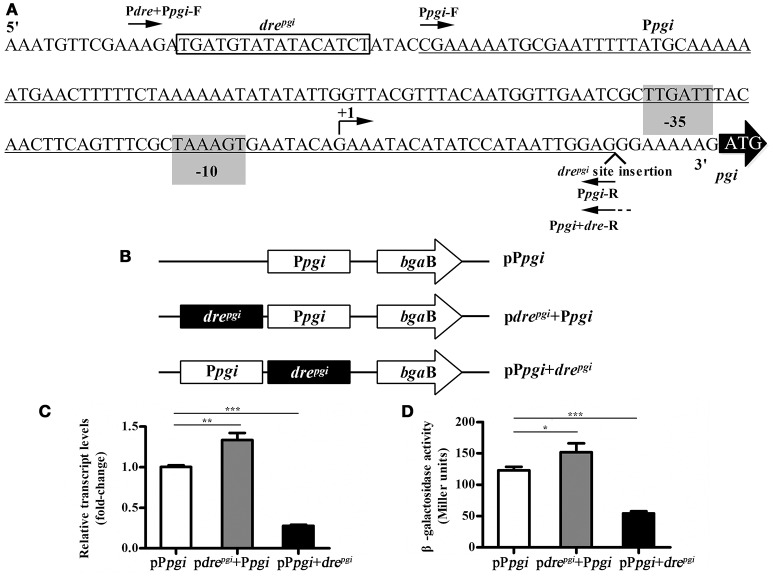
Influence of the relative position of the promoter and the *dre* site in *pgi* gene. **(A)** Binding site of NagR_Bt_ and promoter in the upstream region of *pgi* gene. The putative−35 and−10 positions (predicted by Neural Network Promoter Prediction, http://www.fruitfly.org/seq_tools/promoter.html, score 0.94) are shaded. The corresponding predicted +1 is indicated by a bent arrow and the *dre* site is boxed. The *pgi* promoter region (underlined) was amplified with primers (P*pgi*-F and P*pgi*-R). The fragment with the *dre* site before the promoter (i.e., native configuration) was amplified with primers (P*dre*+P*pgi*-F and P*pgi*-R) and the fragment with *dre* site after the promoter with primers (P*pgi*-F and P*pgi*+*dre*-R). **(B)** Schematic diagram of the change in the relative position with promoter and *dre* site. **(C)** qRT-PCR was used to examine the expression of *bgaB* in three plasmids (p*dre*^*pgi*^+P*pgi*, pP*pgi*+*dre*^*pgi*^, and pP*pgi*) in wild-type strain Bti75. **(D)** β-galactosidase activity assay of three plasmids (p*dre*^*pgi*^+P*pgi*, pP*pgi*+*dre*^*pgi*^, and pP*pgi*) in wild-type strain Bti75. Significance was calculated by two-sample *t*-test (*p < 0.05, **p < 0.01, and ***p < 0.001).

To further verify this, we chose the upstream region of the *pgi* gene (Figure 4A) to construct the corresponding plasmids. The promoter-probe vector was used to detect the influence of changing the relative position of the promoter and the *dre* site on the expression of a downstream reporter gene (Figure 4B). The upstream sequence of *pgi* was selected to construct corresponding plasmids, and then transferred into Bti75. The qRT-PCR analysis showed (Figure 4C) that the expression of *bgaB* was ~1.3-fold increase in p*dre*^*pgi*^+P*pgi* and 0.28-fold decrease in pP*pgi*+*dre*^*pgi*^ compared with that in pP*pgi* respectively. In addition, the corresponding regulatory sequences were analyzed by evaluating β-galactosidase activity (Figure 4D). The result showed that the trend of β-galactosidase activity was consistent with qRT-PCR results.

### Consensus sequence of NagR_Bt_-responsive elements (*dre*) in Bti75

**Figure 5:**
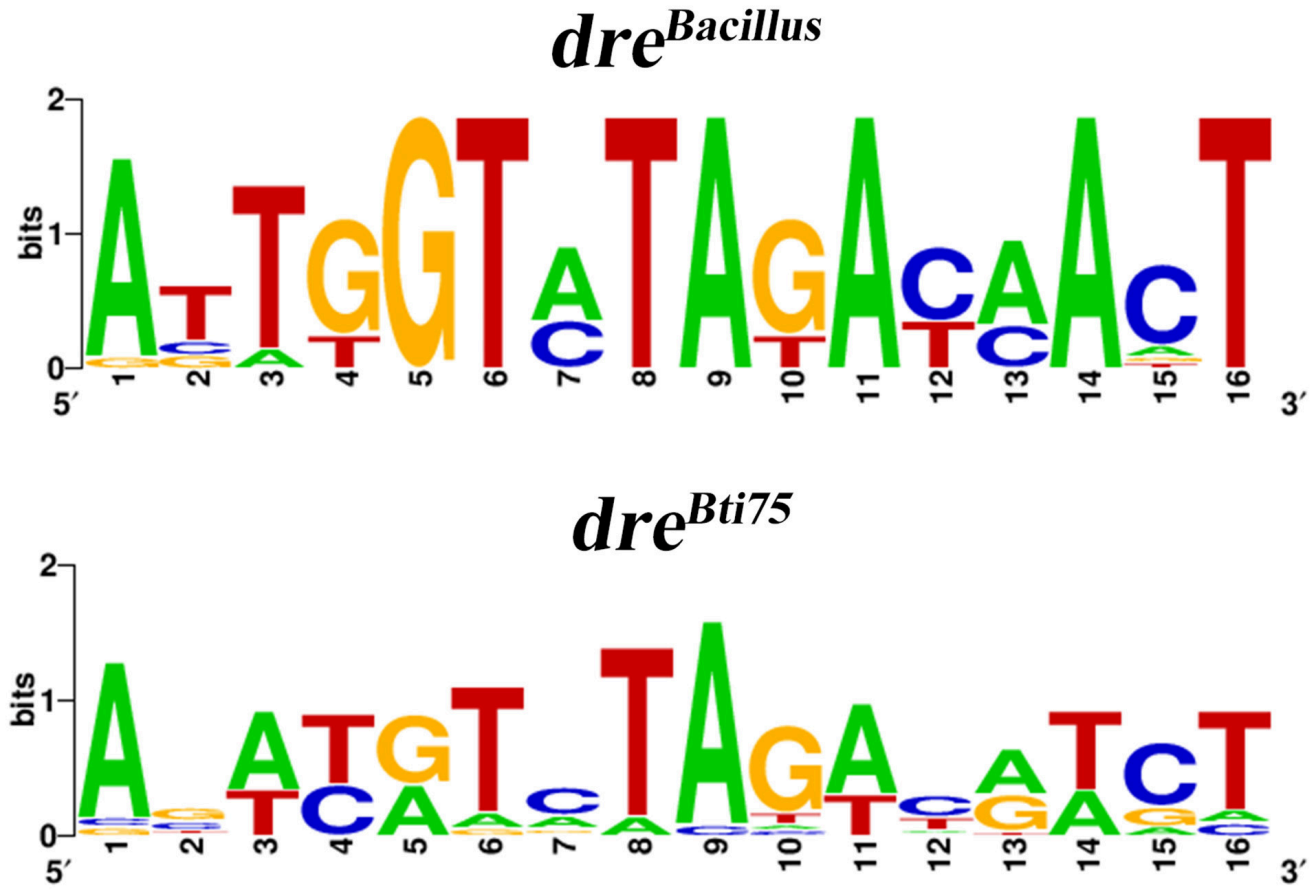
WebLogo representation of *Bacillus* and Bti75 consensus *dre* sites. WebLogo representation of *dre*^Bti75^ was generated with all confirmed NagR_Bt_-binding sequences and the WebLogo representation of *dre*^*Bacillus*^ was generated with the *dre* sites from the upstream regions of the *nagAB* and *nagP* genes in *Bacillus* species (Bertram et al., 2011).

The consensus sequence of *dre* was generated using WebLogo (http://weblogo.berkeley.edu/) (Crooks et al., 2004) based on the NagR_Bt_-binding sequences in Bti75 (Figure 5). The overall height of the stack indicates the sequence conservation at that position, while the height of symbols within the stack indicates the relative frequency of each nucleic acid at that position. Compilation of known and newly identified NagR_Bt_-binding sites led us to construct a NagR_Bt_ consensus sequence. It is not a perfect palindrome compared with previous graphical representations of *Bacillus dre* sites.

## Discussion

**Figure 6:**
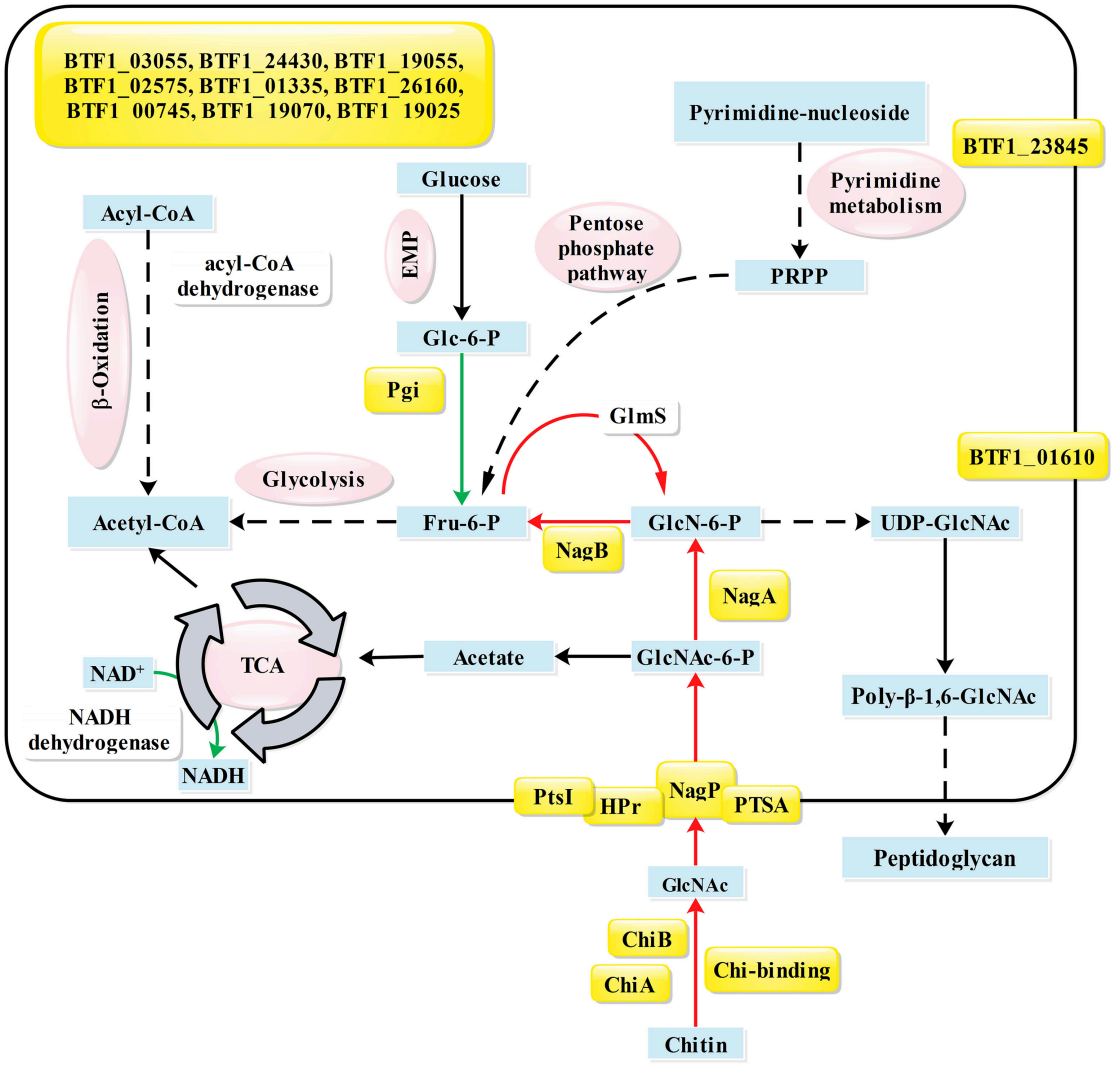
Summary of pathways and genes influenced by NagR_Bt_. Proteins whose genes have a NagR_Bt_-binding site in their upstream region are indicated by yellow shading. Arrows in green or red show genes whose expression is upregulated or downregulated, respectively, in the Δ*nagR* mutant compared with their expression in the wild-type strain Bti75. Key metabolites are shown in blue boxes and different metabolic pathways are indicated in magenta ovals. Gene annotations are based on the KEGG database: BTF1_00220, *nagP*; BTF1_01610, outer surface protein; BTF1_11665, *lpmo*; BTF1_16665, *chiA*; BTF1_18555, *ptsI*; BTF1_18560, *ptsH*; BTF1_18585, *nagB*; BTF1_18590, *nagA*; BTF1_19070, hypothetical protein; BTF1_24980, *crr*; BTF1_28050, *chiB*; BTF1_19025, hypothetical protein; BTF1_23020, *pgi*; BTF1_00745, lytR family protein; BTF1_01335, peptidase; BTF1_02575, major facilitator superfamily protein; BTF1_03055, TetR family transcriptional regulator; BTF1_19055, *pepT*; BTF1_23845, wall-associated protein; BTF1_24430, *chbB*; BTF1_26160: *bofA*; PRPP, 5-phosphoribosyl diphosphate.

In *B. subtilis*, NagR only directly represses the *nagP* and *nagAB* genes (Bertram et al., 2011). According to the phylogenetic analysis (Figure S2), NagR_Bt_ is an ortholog to NagR in *Bacillus* species. However, previous studies had identified five NagR_Bt_-binding sites in Bti75 (Jiang et al., 2015), suggesting that the regulation of NagR_Bt_ is broader than NagR_Bs_. To identify new NagR_Bt_-binding sites, we exploited the PREDetector software program to build a matrix model to scan for potential binding sites in the complete genome of Bt HD-789, which is available from the GenBank database (Benson et al., 2007). To gain a better understanding of regulatory factor NagR_Bt_, the transcriptomes of Bti75-Δ*nagR* and wild-type Bti75 in the logarithmic phase were compared to identify potential target genes of *nagR*. To confirm the interaction of NagR_Bt_ with the newly identified *dre* sequences, we tested the *dre*-like sequences using EMSAs. The differentially expressed genes and confirmed *dre* sequences are listed together in Table 2. The main NagR_Bt_-related pathways are summarized in Figure 6. Three different groups of NagR_Bt_-influenced genes are distinguished according to their expression patterns.

Group 1 contains the upregulated genes that are divided into two categories: those that contain a *dre* sequence (verified by EMSAs) and those without a *dre* sequence. *nagAB* (BTF1_18590 and BTF1_18585) and *nagP* (BTF1_00220) in amino sugar metabolism have *dre* sequences and were upregulated in Bti75Δ*nagR* according to the *in silico*, in *vitro*, and in *vivo* results. These results were consistent with the reports that *nagAB* and *nagE2* are repressed by DasR in *streptomycetes* (Rigali et al., 2006; Li et al., 2008; Nothaft et al., 2010) and by NagR in *B. subtilis* (Bertram et al., 2011). From the transcriptome data, *nagR* deletion resulted in changes to the expression levels of other genes. Subsequently, *dre*-like sites were identified in the corresponding upstream regions using PREDetector and verified using EMSAs, and suggested that NagR_Bt_ could directly influence their expression.

Among these genes, *chiA* (BTF1_16665), *chiB* (BTF1_28050), and chitin-binding protein gene (BTF1_11665) in chitin metabolism are negatively regulated by NagR_Bt_, as confirmed using RNA-seq. This was consistent with the results obtained by Jiang et al. (2015), who reported that *chiB* is regulated not only by CcpA_Bt_ but also by NagR_Bt_. Similarly, the promoter region of chitinase gene contains a W-box binding element that is specifically bound by WRKY, a class of transcription factors in plants (Gao et al., 2014). This suggested that the expression of *chiB* is strictly regulated by multiple transcription factors.

A close inspection of the promoter regions revealed *dre*-binding sites upstream of some phosphotransferase (PTS) system-related genes, such as *crr* (BTF1_24980), *chbBC* (BTF1_24430, BTF1_24425), and *ptsH* (BTF1_18560). This result was consistent with the conclusion of Rigali et al. (2004), demonstrating that the sugar phosphotransferase system is regulated by DasR regulator in *S. coelicolor* (*in vitro* and *in vivo* experiments).

The other subcategory included upregulated genes that did not have *dre* sequences, such as cytidine deaminase (BTF1_06850), pyrimidine-nucleoside phosphorylase (BTF1_06845), nucleoside transporter *nupC* (BTF1_06840) involved in pyrimidine metabolism, glucosamine-fructose-6-phosphate aminotransferase, *glmS* (BTF1_26825) involved in amino sugar metabolism, and so on. These genes are believed to be indirectly regulated by NagR_Bt._ For example, the expression of *glmS* (BTF1_26825) and its riboswitch gene (BTF1_26820) showed increased expression in this study. The enzyme glutamine-fructose-6-phosphate amidotransferase, encoded by the *glmS* gene, catalyzes the reaction between fructose-6-phosphate (Fru-6-P) and glutamine to generate glucosamine-6-phosphate (GlcN-6-P) (Plumbridge et al., 1993). This reaction usually occurs in the process of cell wall biosynthesis, which involves the production of UDP-GlcNAc (Komatsuzawa et al., 2004). We speculated that the inactivation of the *nagR* gene relieved its inhibitory effect on *nagAB*, resulting in their increased expression. GlcN-6-P deaminase, the *nagB* gene product, converts GlcN-6-P to Fru-6-P and can decrease the intracellular GlcN-6-P level in the absence of an exogenous amino sugar supply. It has been reported that GlcN-6-P-induced riboswitch self-cleavage is coupled to the intracellular stability of the *glmS* transcript (Winkler et al., 2004; Collins et al., 2007). When the content of GlcN-6-P is below a certain threshold, the riboswitch is not activated. Consequently, *glmS* transcripts increase to maintain a balanced level of intracellular GlcN-6-P.

The genes of group 2 are not differentially expressed between the Δ*nagR* mutant strain and the wild-type but possess specific *dre* sequences that are identified by EMSAs. Some researchers have reported that transcription factors bind thousands of genomic locations in the vicinity of both active and inactive regions in eukaryotes (Li et al., 2008; Biggin, 2011). A number of weakly bound sites detected this way failed to drive transgenic reporter expression (Fisher et al., 2012). These findings lead to the notion of “non-functional” or “spurious” binding. However, some of the apparently “non-functional” or “spurious” binding sites might turn out to play pivotal roles in maintaining adequate transcriptional regulation under the condition of genetic or environmental abnormalities (Spivakov, 2014). In addition, an increasing number of examples of transcription factor binding sites are not associated with specific gene regulation in prokaryotes (Grainger et al., 2005; Qian et al., 2016). Eight genes have recognized *dre* sites but are not differentially expressed; we speculated that some of them might be involved in the type of specific non-functional interactions, such as gene *bofA* (BTF1_26160). Its gene product, BofA, exerts negative regulation by controlling the level of the SpoIVEA protein in pro-σ^K^ processing during sporulation (Resnekov, 1999; Zhou and Kroos, 2004). We speculated that *bofA* itself barely expresses in the exponential growth stage and is only activated in the process of sporulation. This suggested that NagR_Bt_ might have spatiotemporal influence, for instance in the mother cell during the late stages of sporulation, to regulate the expression of other genes. This aspect of NagR_Bt_ regulation requires further research.

Another gene, *tetR* (BTF1_03055), is one of the TetR family of regulators, members of which regulate genes involved in carbon metabolism, amino acid metabolism, signal transduction systems, antibiotic production, and other physiological metabolism (Ramos et al., 2005; Cuthbertson and Nodwell, 2013). Bioinformatics analysis showed that only one member of the TetR family of regulators has a *dre*-binding site in its upstream promoter region. However, the expression level of *tetR* was unchanged after deletion of *nagR*, as determined by qRT-PCR. A plausible explanation is that there might be another more powerful transcription factor than NagR_Bt_ that also regulates *tetR*. Some suspected transcription factors, such as SigL and Xre, which were found by using DBTBS prediction (Sierro et al., 2008), might play a role in regulating *tetR*. Verification of the connections among these regulators requires further experimental support.

The genes in group 3 are downregulated in the Δ*nagR* strain and are thus believed to be indirectly regulated by NagR_Bt_ because most GntR/HutC family regulators are repressors. Most of the 12 downregulated genes lacking binding sites are involved in fatty acid metabolism. Surprisingly, the EMSA experiments revealed that two of these genes (BTF1_19025 and BTF1_23020) were downregulated and had *dre* sites. Moreover, qRT-PCR showed that the expression levels of both genes were downregulated in the Δ*nagR* strain compared with that in the wild-type. Together, our findings highlight that NagR_Bt_ can activate these two genes. The gene of BTF1_19025 encodes a hypothetical protein belonging to a conserved protein domain family (DUF3932) and proteins in this family are functionally uncharacterized by searching NCBI's Conserved Domain Database (CDD) (Marchler-Bauer et al., 2017). The *pgi* gene (BTF1_23020) encodes the enzyme glucose-6-phosphate isomerase that participates in many pathways in the interconversion of glucose 6-phosphate and fructose-6-phosphate. The *dre* sites in these two genes were located upstream of the predicted promoter region just as in the classic activation mechanism in bacteria (Lee et al., 2012). It has been reported that the different functions (promotion or inhibition) of CcpA are caused by the different position of the *cre* sites in each gene (Deutscher et al., 2006). When the *cre* site of major genes is located downstream of the promoter, gene expression is often repressed by the CcpA complex. However, if the *cre* site is located upstream of the promoter, the CcpA complex stimulates the transcription to activate gene expression, such as in *ackA* (Moir-Blais et al., 2001), *pta* (Presecan-Siedel et al., 1999), *ilv*-*leu* (Shivers and Sonenshein, 2005; Tojo et al., 2005), and *pepQ* (Luesink et al., 1998). Other examples of dual function transcription activators and repressors are known in bacteria e.g., Cra and Crp (Pérez-Martín and de Lorenzo, 1997; Cozzone, 1998).

To further confirm the activation role of NagR_Bt_, we constructed a shuttle promoter-probe vector incorporating the thermostable gene *bgaB* with the promoter of *pgi*. qRT-PCR revealed that the expression of *bgaB* was ~1.3-fold increased by *dre*+P*pgi* compared with P*pgi* and ~0.28-fold decreased by P*pgi*+*dre* compared with P*pgi*. Furthermore, the trend of β-galactosidase activity was consistent with qRT-PCR results. It demonstrates effectively that a downstream *dre* site acts as an effective repressor, whereas its inactivation produces a relatively small decrease in *pgi* expression. So far, there has been no report of this gene being directly regulated by NagR_Bt_; it has been reported this gene was regulated by CcpA in *Streptococcus suis* (Willenborg et al., 2014).

In addition, the NagR_Bt_-binding site consensus sequence was generated by compiling known and newly identified binding sites using the *in silico, in vitro*, and *in vivo* data (Figure 5). The consensus sequence of the NagR_Bt_-binding site is not a perfect palindrome and is different from the *Bacillus* species *dre* sites (Bertram et al., 2011). It has obvious conserved recognition sites at positions 5, 6, 8, 9, 11, 14, and 16 in *dre*^*Bacillus*^, but the bases in the same position are not strictly conserved in *dre*^Bti75^. To avoid the omission of NagR_Bt_-binding sites, our future research will include the use of chromatin immunoprecipitation sequencing.

Compared with the wide-ranging regulation by DasR in *Streptomycetes* (Swiatek-Polatynska et al., 2015), where hundreds of *dre*-binding sites have been identified, NagR_Bt_-binding sites have a narrower distribution and are found in genes encoding proteins involved in amino sugar metabolism, the phosphotransferase system, chitin metabolism, and other pathways. By contrast, NagR_Bs_-binding sites are limited to the *nagP* and *nagAB-nagR* locus. This suggests that although NagR_Bt_ is a less prominent regulator than DasR, its regulation is more widespread than that of NagR_Bs_.

Overall, our results demonstrated that NagR_Bt_ functions as an important regulator of metabolism, not only by directly regulating the amino sugar metabolism and phosphotransferase system, but also by directly or indirectly influencing other biological processes, such as nucleotide metabolism, fatty acid metabolism, and the EMP pathway. In addition, NagR_Bt_ is not only a pleiotropic transcriptional regulator but also a dual transcription regulator that acts as both a repressor and an activator. This adds to our understanding of the role of the GntR/HutC family transcription factors.

## Author contributions

ZC and JC designed the research. ZC performed the experimental work and drafted the manuscript. TT performed the RNA-seq analysis. YZ and LH constructed *nagR*-complemented strain and carried out promoter/*dre* position swapping experiments. XH purified NagR_Bt_ protein. HM performed the β-galactosidase assay. All authors read and approved the final manuscript.

### Conflict of interest statement

The authors declare that the research was conducted in the absence of any commercial or financial relationships that could be construed as a potential conflict of interest.
