# Supplementary material for: NagRBt Is a Pleiotropic and Dual Transcriptional Regulator in Bacillus thuringiensis
Source: Front Microbiol. 2018 Sep 11;9:1899. doi: 10.3389/fmicb.2018.01899 (PMC6141813; doi:10.3389/fmicb.2018.01899)
Supplement: Supplementary file 2 [file Table_3.DOC]

Supplementary Material

# NagR_Bt_ is a pleiotropic and dual transcriptional regulator in *Bacillus thuringiensis*

**Zhang-lei Cao^1^, Tong-tong Tan^1^,** **Yan-li Zhang^1^, Lu Han^1^, Xiao-yue Hou^1^, Hui-yong Ma^1^,** **Jun Cai^1,2,3^***

^1^Department of Microbiology, College of Life Sciences, Nankai University, Tianjin, China;

^2^Key Laboratory of Molecular Microbiology and Technology, Ministry of Education Tianjin, China;

^3^Tianjin Key Laboratory of Microbial Functional Genomics, Tianjin, China

*** Correspondence:**Corresponding Author: Jun Cai
caijun@nankai.edu.cn

# Supplementary Figures and Tables

## Supplementary Figures


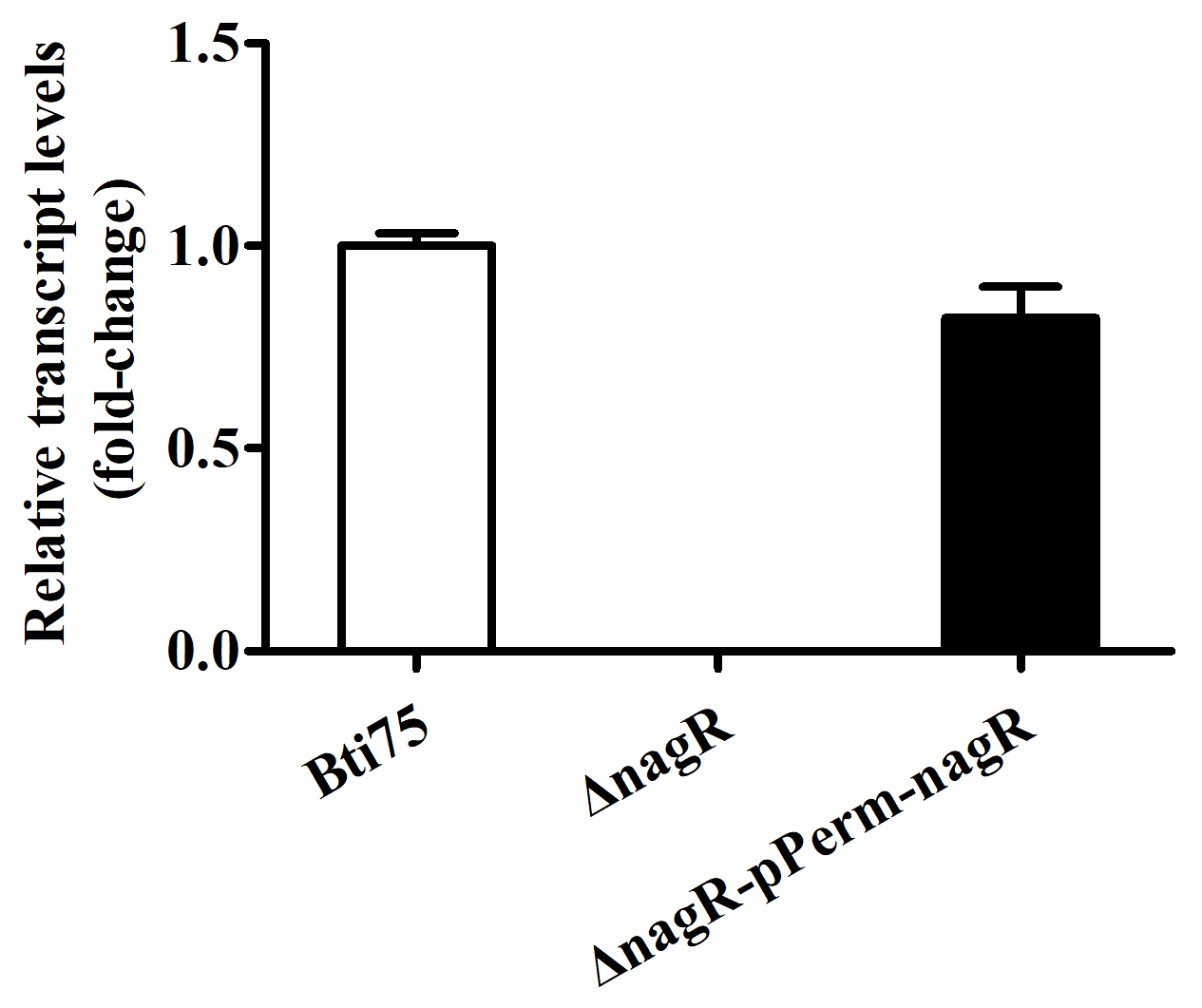


**Supplementary Figure 1.** The expression of gene *nagR* in wild-type strain, Bti75*ΔnagR* and Bti75*ΔnagR*-pP*erm*-*nagR*.


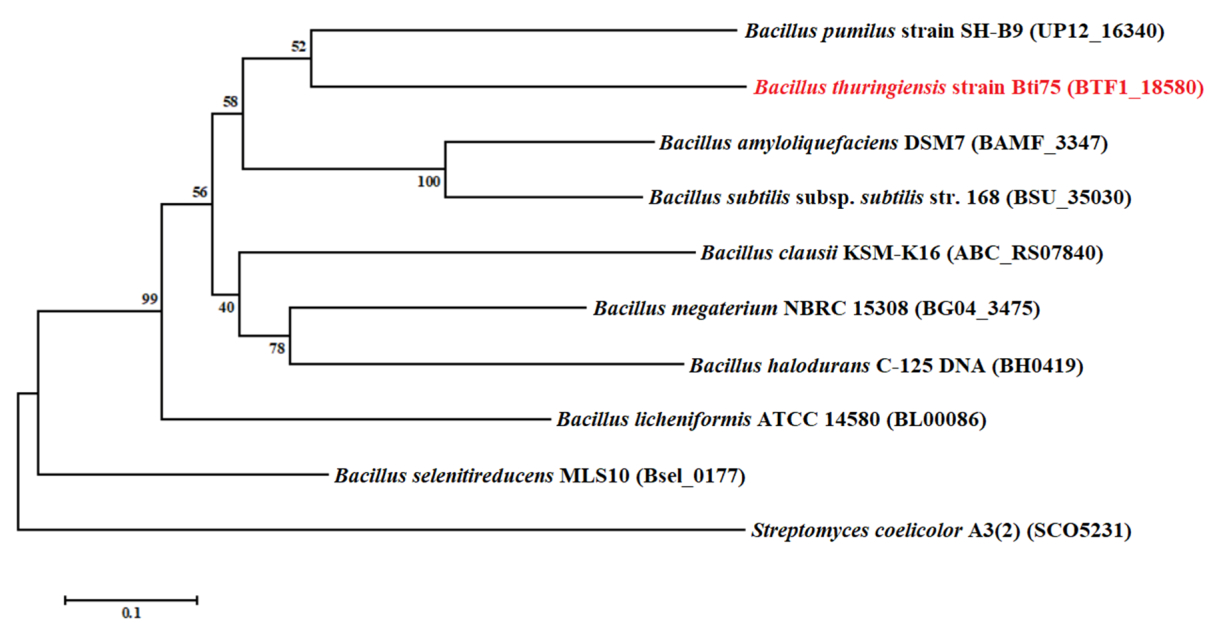


**Supplementary Figure 2.** Phylogenetic analysis of NagR regulators from *Bacillus* species and DasR from *S. coelicolor*. Accession numbers of *nagR* gene are shown in parentheses.

## Supplementary Tables

**Supplementary Table 2.** Synthetic primers used in study.

Table S1 Synthetic primers used in study.

| Primer | Sequence (5’→3’) | Function |
| --- | --- | --- |
| P*erm*-F | CTGGTCGACGCAAACTTAAGAGTGTGT | P*erm*-cloning |
| P*erm*-R | GAATACTTGTCGATGTTCATGTAATTACTCCTGAAGTGA | P*erm*-cloning |
| *nagR*-F | ACTTCAGGAGTAATTACATGAACATCGACAAGTATTCACC | *nagR*-cloning |
| *nagR*-R | AGATCTAGACTATTTGTTACGTGCAATATTC | *nagR*-cloning |
| P*pgi*-F | CCGGATCCCGAAAAATGCGAATTTTTATGC | *pgi* position swap experiment |
| P*pgi*-R | CCGTCGACCTCCAATTATGGATATGTATTTCTG | *pgi* position swap experiment |
| P*dre*+P*pgi*-F | CCGGATCCAAGATGATGTATATACATCTATAC | *pgi* position swap experiment |
| P*pgi*+*dre*-R | GCGTCGACGTATAGATGTATATACATCATCTTCTCCAATTATGGATATGTATTTCTG | *pgi* position swap experiment |
| *ptsA*-EF | TTTTTTTGCCCAACTTGTCTAGAGGTCTTTACTACATTTT | *ptsA*-EMSA |
| *ptsA*-ER | AAAATGTAGTAAAGACCTCTAGACAAGTTGGGCAAAAAAA | *ptsA*-EMSA |
| *ptsA*-ER(B) | AAAATGTAGTAAAGACCTCTAGACAAGTTGGGCAAAAAAA(5’ biotin) | *ptsA*-EMSA |
| *chiA*-EF | AACCAATCATCTAGACAACTATATAACACGAACGTTTTAC | *chiA*-EMSA |
| *chiA*-ER | GTAAAACGTTCGTGTTATATAGTTGTCTAGATGATTGGTT | *chiA*-EMSA |
| *chiA*-ER(B) | GTAAAACGTTCGTGTTATATAGTTGTCTAGATGATTGGTT(5’ biotin) | *chiA*-EMSA |
| 11665-EF | GAAAATAACTATAGTTGGCTAGTCATCTTCTCTACATTTA | 11665-EMSA |
| 11665-ER | TAAATGTAGAGAAGATGACTAGCCAACTATAGTTATTTTC | 11665-EMSA |
| 11665-ER(B) | TAAATGTAGAGAAGATGACTAGCCAACTATAGTTATTTTC(5’ biotin) | 11665-EMSA |
| *nagA*-EF | ATCTTAATATATGCACGAGTAGTTGTCTATACATTCTAAT | *nagA*-EMSA |
| *nagA*-ER | ATTAGAATGTATAGACAACTACTCGTGCATATATTAAGAT | *nagA*-EMSA |
| *nagA*-ER(B) | ATTAGAATGTATAGACAACTACTCGTGCATATATTAAGAT(5’ biotin) | *nagA*-EMSA |
| 01610-EF | AGATGTAAAGTATAGATGTATAGACTTGTAGGTGTTAAGA | 01610-EMSA |
| 01610-ER | TCTTAACACCTACAAGTCTATACATCTATACTTTACATCT | 01610-EMSA |
| 01610-ER(B) | TCTTAACACCTACAAGTCTATACATCTATACTTTACATCT(5’ biotin) | 01610-EMSA |
| *nagP*-EF | AAAATAAAAACTACACATCTATACAACTATATGTTATTAT | *nagP*-EMSA |
| *nagP*-ER | ATAATAACATATAGTTGTATAGATGTGTAGTTTTTATTTT | *nagP*-EMSA |
| *nagP*-ER(B) | ATAATAACATATAGTTGTATAGATGTGTAGTTTTTATTTT(5’ biotin) | *nagP*-EMSA |
| *ptsH*-EF | CAGTATTTTTTTAGTTGTATAGACGTGAGTACTACATCAT | *ptsH*-EMSA |
| *ptsH*-ER | ATGATGTAGTACTCACGTCTATACAACTAAAAAAATACTG | *ptsH*-EMSA |
| *ptsH*-ER(B) | ATGATGTAGTACTCACGTCTATACAACTAAAAAAATACTG(5’ biotin) | *ptsH*-EMSA |
| 19070-EF | ATGGTTATGTACACATGTCAAGACAACTTATTACAATAGA | 19070-EMSA |
| 19070-ER | TCTATTGTAATAAGTTGTCTTGACATGTGTACATAACCAT | 19070-EMSA |
| 19070-ER(B) | TCTATTGTAATAAGTTGTCTTGACATGTGTACATAACCAT(5’ biotin) | 19070-EMSA |
| *pgi*-EF | AAATGTTCGAAAGATGATGTATATACATCTATACCGAAAA | *pgi*-EMSA |
| *pgi*-ER | TTTTCGGTATAGATGTATATACATCATCTTTCGAACATTT | *pgi*-EMSA |
| *pgi*-ER(B) | TTTTCGGTATAGATGTATATACATCATCTTTCGAACATTT(5’ biotin) | *pgi*-EMSA |
| 19025-EF | AACCAATCATCTAGACAACTATATAACACGAACGTTTTAC | 19025-EMSA |
| 19025-ER | GTAAAACGTTCGTGTTATATAGTTGTCTAGATGATTGGTT | 19025-EMSA |
| 19025-ER(B) | GTAAAACGTTCGTGTTATATAGTTGTCTAGATGATTGGTT(5’ biotin) | 19025-EMSA |
| 03055-EF | ATACAAAAGTATAGATGTATAGTCGACTTTGTGTATTTAA | 03055-EMSA |
| 03055-ER | TTAAATACACAAAGTCGACTATACATCTATACTTTTGTAT | 03055-EMSA |
| 03055-ER(B) | TTAAATACACAAAGTCGACTATACATCTATACTTTTGTAT(5’ biotin) | 03055-EMSA |
| 19055-EF | TAGAAAGCAGATGGTTATCTAGACATGTAAACCATCTGTT | 19055-EMSA |
| 19055-ER | AACAGATGGTTTACATGTCTAGATAACCATCTGCTTTCTA | 19055-EMSA |
| 19055-ER | AACAGATGGTTTACATGTCTAGATAACCATCTGCTTTCTA(5’ biotin) | 19055-EMSA |
| 23845-ER | GGTTTTATTTTTAGATGTATAGATGACGAGACTAAAATAT | 23845-EMSA |
| 23845-EF | ATATTTTAGTCTCGTCATCTATACATCTAAAAATAAAACC | 23845-EMSA |
| 23845-ER(B) | ATATTTTAGTCTCGTCATCTATACATCTAAAAATAAAACC(5’ biotin) | 23845-EMSA |
| 02575-EF | CATATCATATCTAGTTATTTAGATATCTGTCTTTGTATTT | 02575-EMSA |
| 02575-ER | AAATACAAAGACAGATATCTAAATAACTAGATATGATATG | 02575-EMSA |
| 02575-ER(B) | AAATACAAAGACAGATATCTAAATAACTAGATATGATATG(5’ biotin) | 02575-EMSA |
| 01335-EF | ACTATAATAAAATTACAACTATACAACTAGAGGAGGTCTC | 01335-EMSA |
| 01335-ER | GAGACCTCCTCTAGTTGTATAGTTGTAATTTTATTATAGT | 01335-EMSA |
| 01335-ER(B) | GAGACCTCCTCTAGTTGTATAGTTGTAATTTTATTATAGT(5’ biotin) | 01335-EMSA |
| 26160-EF | TTGTCTTGTCCCATACAAATAGATGTATAGGTCATTCATA | 26160-EMSA |
| 26160-ER | TATGAATGACCTATACATCTATTTGTATGGGACAAGACAA | 26160-EMSA |
| 26160-ER(B) | TATGAATGACCTATACATCTATTTGTATGGGACAAGACAA(5’ biotin) | 26160-EMSA |
| 00745-EF | ATGTGTTTGAATACACTTCTAAATGTATATACTTCGATAG | 00745-EMSA |
| 00745-ER | CTATCGAAGTATATACATTTAGAAGTGTATTCAAACACAT | 00745-EMSA |
| 00745-ER(B) | CTATCGAAGTATATACATTTAGAAGTGTATTCAAACACAT(5’ biotin) | 00745-EMSA |
| 16S-F(QP) | CCGCGGTAATACGTAGGTG | qRT-PCR |
| 16S-R(QP) | TTTCCAATGACCCTCCACG |  |
| *bgaB*-F(QP) | TGGAGCAAGATCGAACCGTC | qRT-PCR |
| *bgaB*-R(QP) | AGCTGGAGTAGTTGCAGTCG |  |
| *cdd*- F(QP) | AACTGCGCAGAAAGAACAGC | qRT-PCR |
| *cdd*-R(QP) | CCACATGGTGAAATCGGTGC |  |
| 19025-F(QP) | GTCTGATCACCCAGCACA | qRT-PCR |
| 19025-R(QP) | GTAGTTGAAACGCGTTGG |  |
| 01610-F(QP) | TCCGCATCGTTATTCAGGGTT | qRT-PCR |
| 01610-R(QP) | AAGCAGCCGTTCTCATACCG |  |
| *nagP*-F(QP) | CTTCGCTGGAAAACGTTTCGT | qRT-PCR |
| *nagP*-R(QP) | ACCGTGTGACACTGCATCAA |  |
| *chiA*-F(QP) | ACTACAATGCTGGTAGCGG | qRT-PCR |
| *chiA*-R(QP) | CGAACGAAGAGCTGGGAAA |  |
| *ptsH*-F(QP) | AGACATTACGCCCATGATTGA | qRT-PCR |
| *ptsH*-R(QP) | CTCGTCCAGCAACTCTAC |  |
| 19070-F(QP) | TTGTTTCTGCTGAAATGCTCCA | qRT-PCR |
| 19070-R(QP) | TCTTGTAGCGATTGTATGTATGGC |  |
| *crr*-F(QP) | GCTTGGCCTTCAGAAACGTG | qRT-PCR |
| *crr*-R(QP) | AAGCGAAAAACGGCACAGAA |  |
| 08960-F(QP) | AAGGTTCTTCAACGGCGACT | qRT-PCR |
| 08960-R(QP) | AGCTACGTGATGCCCTTTCC |  |
| 20050-F(QP) | TCGATGCCACTTGTCCAGATG | qRT-PCR |
| 20050-R(QP) | AGGTGAACGATGTCAGGTGC |  |
| *pgi*-F(QP) | GTGGGTAGACCTTCCGCTTC | qRT-PCR |
| *pgi*-R(QP) | CGCGTGCCCCTAAGTAAGAA |  |
| 12180-F(QP) | AATGGCCTCCTAAGCGATGT | qRT-PCR |
| 12180-R(QP) | GGGTGGTTCGTCCGAGTAAG |  |
| 24870-F(QP) | AGGATGTGCCGACTCTTGTG | qRT-PCR |
| 24870-R(QP) | ATCCGTCGCATGTAGCTCTG |  |
| *nheA*-F(QP) | AGGGGCAAACGGAAGTGAAA | qRT-PCR |
| *nheA*-R(QP) | AGCGGTGATTGTGATCCTAAC |  |
| 26870-F(QP) | TGTGGGTGGAAAAATTGCTGG | qRT-PCR |
| 26870-R(QP) | CCTTGAGTCGCATTCATTCCG |  |
| *nagA*-F(QP) | TGCACCTGCACGTTTCTTTG | qRT-PCR |
| *nagA*-R(QP) | ATGACACAAGCTCCAGAGG |  |
| *nagR*-F(QP) | GCTGGAACTAGGCGGAAACT | qRT-PCR |
| *nagR*-R(QP) | CGGAAGCGTGGAATTGGAAC |  |
| *nprE*-F(QP) | CAGGGGCGTTAAATGAAGCG | qRT-PCR |
| *nprE*-R(QP) | GAAGCGCGTCTCCTGCTTTAC |  |
|  |  |  |

**Supplementary Table 2.** The predicted NagR_Bt_ binding sites identified by PREDetector with the cut-off score 7.0 according to the corresponding 16-bp *dre* sequence of *nagA*, *nagP*, *chiA*, *chiB* and *chitin*.

The data is in Table S2, XLS file.
